# Dosimetric consequences of adapting the craniocaudal isocenter distance to daily patient position in craniospinal irradiation using volumetric modulated arc therapy

**DOI:** 10.1002/acm2.14530

**Published:** 2024-10-24

**Authors:** Annele Heikkilä, Antti Vanhanen, Maija Rossi, Tuomas Koivumäki, Michiel Postema, Eeva Boman

**Affiliations:** ^1^ Department of Biomedical Technology, Faculty of Medicine and Health Technology Tampere University Tampere Finland; ^2^ Department of Medical Physics, Tampere University Hospital Wellbeing Services County of Pirkanmaa Tampere Finland; ^3^ Department of Oncology, Tampere University Hospital Wellbeing Services County of Pirkanmaa Tampere Finland; ^4^ Department of Medical Physics, Hospital Nova of Central Finland Wellbeing Services County of Central Finland Jyväskylä Finland; ^5^ School of Electrical and Information Engineering University of the Witwatersrand, Johannesburg Braamfontein South Africa

**Keywords:** craniospinal radiotherapy, junction setup errors, partial‐arc VMAT, robustness of CSI

## Abstract

**Purpose:**

In craniospinal irradiation, two or three isocenter groups along the craniocaudal axis are required to cover the long treatment target. Adapting the isocenter distance according to daily deviations in patient position is challenging because dosimetric hot or cold spots may occur in the field junction. The aim of this study was to quantify the effect of adapting the isocenter distance to patient position on the dose distribution of the field overlap region in craniospinal irradiation using partial‐arc volumetric modulated arc therapy.

**Methods:**

The magnitude of isocenter distance deviations in craniocaudal direction was quantified by registering the setup images of 204 fractions of 12 patients to the planning images. The dosimetric effect of these deviations was determined by shifting the isocenters of the original treatment plan and calculating the resulting dose distribution.

**Results:**

On fraction‐level, deviations larger than 3 mm caused more than 5 percentage point changes in the doses covering 2% (*D*_2%_) and 98% (*D*_98%_) of the junction volume in several patients. On treatment course‐level, the changes in *D*_2%_ and *D*_98%_ of the junction volume were less than 5 percentage points in all cases except for one patient.

**Conclusions:**

Craniocaudal isocenter distance adaptation can be conducted provided that the mean isocenter distance deviation over the treatment course is within 3 mm.

## 1 INTRODUCTION

Craniospinal irradiation, radiotherapy of the brain and spinal canal, is an important treatment modality of medulloblastoma and other malignancies of the central nervous system.^1^, ^2^, ^3^ The long and complex treatment volume exceeds the largest treatment field size of a typical clinical linear accelerator. Thus, several irradiation fields over two or three separate isocenters along the craniocaudal axis must be used. The dose planning of the field junctions between different isocenters is challenging, and patient setup and localization of the treatment fields requires extra care.

Conventionally, three‐dimensional conformal radiotherapy (3D‐CRT) has been used for craniospinal irradiation.^4^ In 3D‐CRT, the radiation dose drops sharply at the edges of the treatment fields. The field junctions are designed so that the field edges are directly adjacent to each other without overlap or gap; otherwise, dosimetric hot or cold spots could develop.^5^ In practice, the craniocaudal distance between the isocenters must be kept constant even if the spine of the patient was stretched or shortened compared to the planning computed tomography (CT) image. This may cause underdosage of the most cranial or caudal part of the target volume. Earlier studies have reported mean residual setup errors of 1.5 and 2.7 mm of the lower spine relative to the brain in craniocaudal direction.^6^, ^7^ In the study by Stoiber et al., the maximum setup error of the lower spine relative to the brain in craniocaudal direction was approximately 11 mm.^7^

Research on craniospinal irradiation has shown that volumetric modulated arc therapy (VMAT) produces a more conformal and homogeneous dose distribution to the treatment volume with less high‐dose radiation exposure to the surrounding organs compared to 3D‐CRT.^8^, ^9^ The downside of VMAT is the spread of low dose to large volumes, which is a concern especially for pediatric patients due to the secondary cancer risk.^10^ The low dose volume can be reduced by using partial arcs for the spinal part of the target volume.^11^, ^12^, ^13^

When optimizing a VMAT plan, it is possible to create a smooth dose gradient in the field junctions by overlapping the radiation fields in craniocaudal direction.^14^, ^15^, ^16^, ^17^, ^18^, ^19^ The smooth dose gradient allows some flexibility in the craniocaudal distance between the isocenters, enabling individual daily anatomy‐based alignment of different isocenter groups.^20^ This might eliminate the need for making compromises in the localization of the different isocenters in craniocaudal direction. However, dosimetric hot or cold spots could result in the field junction if the isocenter distance deviates too much from the plan. Studies assessing the dosimetric impact of intentionally introduced systematic isocenter distance deviations have shown that VMAT plan quality at the field junction is not significantly degraded for isocenter distance deviation up to 3 mm.^14^, ^15^, ^21^, ^22^ In a study utilizing *in‐silico* base dose plans, the plans were robust for isocenter distance deviations up to 5 mm.^18^

The aim of this study was to determine the effect of isocenter‐specific, daily anatomy‐based treatment localization on the field junction dose distribution in craniospinal irradiation using VMAT. Daily setup images of previously treated patients were used to determine the deviation in the craniocaudal isocenter distance. The effect of isocenter distance deviation on daily dose distribution as well as cumulative dose distribution was analyzed. Earlier studies have only simulated the dosimetric effect of systematic isocenter distance deviations.^14^, ^15^, ^16^, ^17^, ^18^, ^19^, ^21^ In clinical practice, however, the setup errors include both random and systematic components. The dosimetric effect of clinically observed daily isocenter distance deviations has not been reported in literature.

## 2 MATERIALS AND METHODS

Twelve unselected consecutive patients who had received craniospinal irradiation during 2014−2022 were retrospectively selected for the study, which was approved by Wellbeing Services County of Pirkanmaa (research permit number R21663). The patient age ranged from 3 to 54 years, with a median age of 16 years. The patients were treated in 10−22 fractions with a prescribed dose of 1.6−2.0 Gy per fraction.

Planning CT images were acquired in supine position with the patients’ arms laying at the sides. The patients’ heads were immobilized with individual thermoplastic masks. The masks of two patients also covered their neck and shoulders. Individual full‐body vacuum bags were used for the fixation of eight patients. Treatment target and organs‐at‐risk were contoured by experienced radiation oncologists. Clinical target volume (CTV) covered the whole brain and spinal canal. Planning target volume (PTV) was created by adding a 5−10 mm margin to CTV. In addition, for four pediatric patients, the vertebral bodies were contoured in PTV to avoid uneven vertebral growth caused by inhomogeneous vertebral dose.^23^ Craniocaudal PTV length varied from 47 to 85 cm.

The patients of this study had originally been treated using either 3D‐CRT or VMAT. The original clinical plans were not used for this study since there were variations in the planning protocols. A new VMAT plan was generated for each patient retrospectively according to the current clinical protocol used in our department. The plans were generated for a TrueBeam linear accelerator in Eclipse (Varian Medical Systems, Palo Alto, California, USA) using 6 MV photon beams. The plans were optimized using Photon Optimizer v. 16.1 and calculated using Acuros XB v. 16.1 with 2.5 mm calculation grid resolution. The multileaf collimator leaf width was 5 mm in the central 40 leaf pairs and 10 mm in the peripheral 20 leaf pairs. The plans were normalized to 100% at PTV mean dose.

**FIGURE 1:**
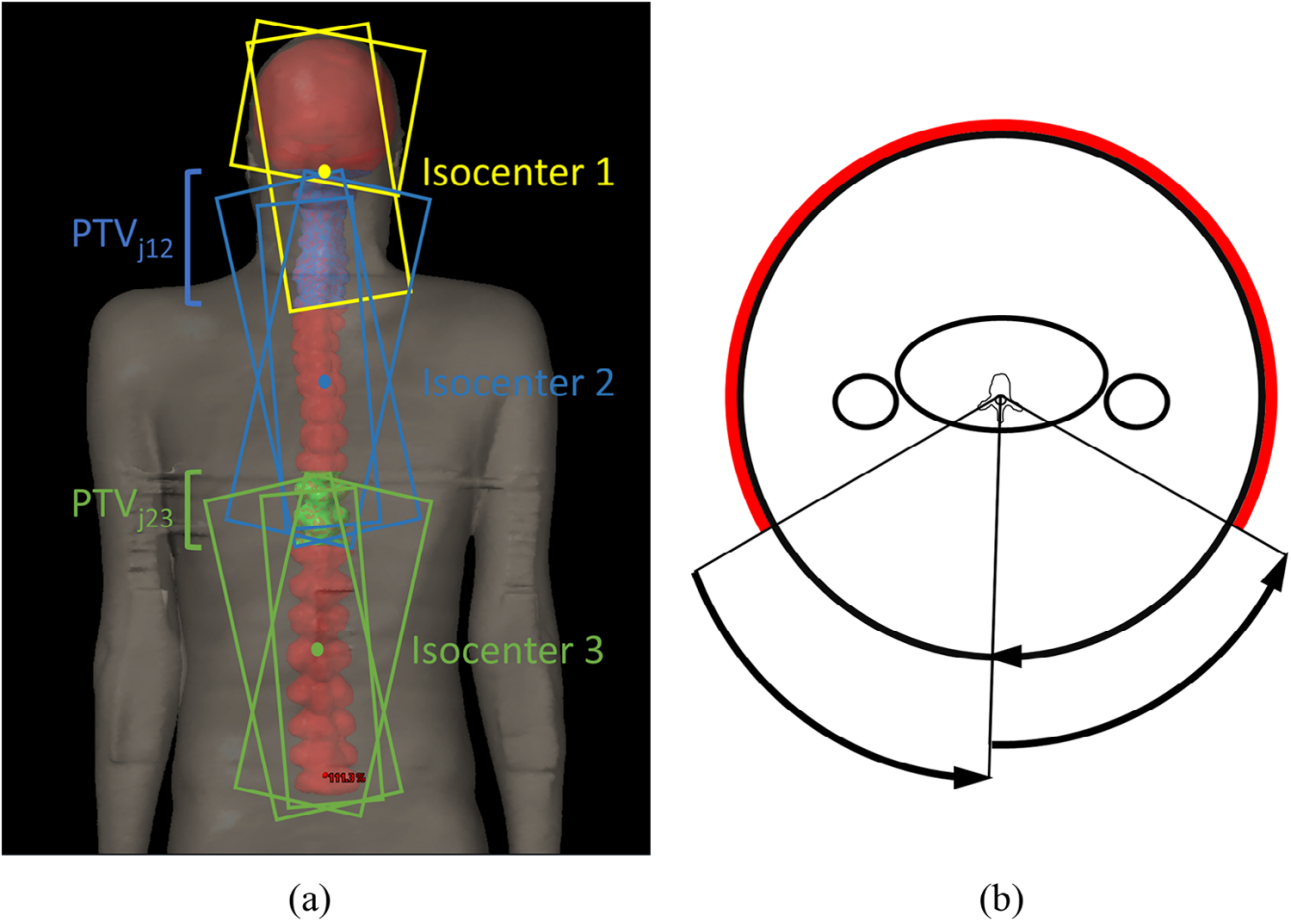
Posterior view of the field arrangement and junction regions (PTV_j12_ and PTV_j23_) for a representative patient with three isocenter groups (a) and an axial view of the field arrangement used for irradiating the spinal canal (b). The start angle of each arc is indicated by a radial line and the end angle is indicated by an arrow. The red arc indicates the avoidance sector.

The treatment plans for patients with PTV length <70 cm (*n* = 5) were generated using two isocenter groups, one for the brain and one for the spine. The treatment plans for patients with PTV length ≥70 cm (*n* = 7) were generated using three isocenter groups, one for the brain and two for the spine. The cranial, upper spinal and lower spinal isocenters were denoted as isocenter 1, isocenter 2, and isocenter 3, respectively (Figure 1a). The plans only had isocenter shifts in the craniocaudal direction. The fields of adjacent isocenter groups were allowed to overlap as much as possible depending on patient size and number of isocenters. The overlap length varied between 7 and 20 cm with median overlap lengths of 13 and 11 cm for the cranial and caudal junction, respectively. The auto‐feathering option of Eclipse was used to guide the optimization algorithm to create a smooth dose gradient over the junction region. In addition, the field edges were staggered by 1−5 cm in craniocaudal direction for most patients. The field junction volumes, PTV_j12_ and PTV_j23_, were defined as the parts of PTV covered by the overlapping fields (Figure 1a). The exact lengths of PTV_j12_ and PTV_j23_ were determined by measuring from the cranial edge of the caudal fields to the caudal edge of the cranial fields at 100 cm distance from the source and adding 1−2 cm to both ends to include dose deviations caused by large setup errors. Corresponding junction regions, CTV_j12_ and CTV_j23_, were defined for CTV.

The brain was irradiated using two 360° arc fields with collimator angles 5°−10° and 80°−85° with isocenter located between the base of the skull and the eyes in the craniocaudal direction. The field with 5°−10° collimator angle also covered the upper part of the cervical spine. The spinal canal was irradiated using posterior partial arc fields. The start and end gantry angles were determined based on the position of the patient's arms so that irradiation through the arms was avoided. Specifically, the spinal canal fields included a right posterior partial arc, a left posterior partial arc and a full arc with avoidance sector covering the anterior side and the arms of the patient (Figure 1b). The radiation dose was delivered to the spine within arc segments of 104°−133° in total. Collimator angles of 4°−10° and 350°−356° were used. The optimization goals for PTV were *V*_95%_ > 95% and *V*_107%_ < 2%, where *V*_X%_ stands for the volume covered by *X*% of the prescribed dose. The dose to organs‐at‐risk, including heart, lungs, kidneys, lenses, bowel and liver, was minimized.

Treatment localization was based on daily image guidance. At each treatment fraction, the patients were initially set up by aligning tattooed marker points to the treatment room lasers. Two‐dimensional kV‐images were acquired at each isocenter position to determine the required setup corrections. For patients that were originally treated using 3D‐CRT (*n* = 6), a lateral image was acquired of the head, and anterior‐posterior images were acquired of the spine. For patients that were originally treated using VMAT (*n* = 6), lateral and anterior‐posterior images were acquired of both the head and the spine. If only translational setup errors or rotational setup errors around the anterior‐posterior axis were detected, the corrections were made using a four‐degrees‐of‐freedom robotic couch, and treatment was delivered. If there were setup errors that could not be corrected using the robotic couch, such as rotations around the craniocaudal or left‐right axes or incorrect curvature of the spine, the patient was manually realigned and new setup images were acquired, until the patient position was corrected as well as possible. In the original treatments, a treatment plan‐based fixed isocenter distance was used in the craniocaudal direction.

For the purpose of this study, craniocaudal setup errors were evaluated separately for each isocenter by registering the setup images acquired after possible manual realignment of the patient to digitally reconstructed radiographs. The cranial setup images were registered to the base of the skull and spinal setup images to the vertebrae. The craniocaudal distance between adjacent isocenters was determined from the couch values of the registered images and compared to the planned isocenter distance. Deviations in isocenter distance were assessed for all delivered fractions for each patient. Group systematic error and standard deviations of the systematic and random error were calculated using the definitions introduced by van Herk.^24^

The effect of the craniocaudal setup error on PTV_j12_ and PTV_j23_ were simulated in separate calculations. First, the effect on PTV_j12_ was simulated by shifting isocenter 2 towards or away from isocenter 1 by the magnitude of the deviation in isocenter distance according to the setup image registration while keeping isocenters 1 and 3 at their planned positions. Next, the effect on PTV_j23_ was simulated by shifting both isocenter 2 and isocenter 3 towards or away from each other by half of the deviation in isocenter distance according to the setup image registration while keeping isocenter 1 at its planned position. Lateral, anterior‐posterior and rotational setup errors were not considered in the simulations. The resulting dose distribution was calculated on the planning CT image. The following dose volume parameters were extracted for the field junction region: mean dose (*D*_mean_), *V*_95%_, *V*_107%_, and doses that covered 98% and 2% of the volume (*D*_98%_ and *D*_2%_).

In addition to the fraction‐specific dose distributions, the cumulative dose distribution for the whole treatment course was calculated by summing up the fraction‐specific dose distributions.

## 3 RESULTS

**FIGURE 2:**
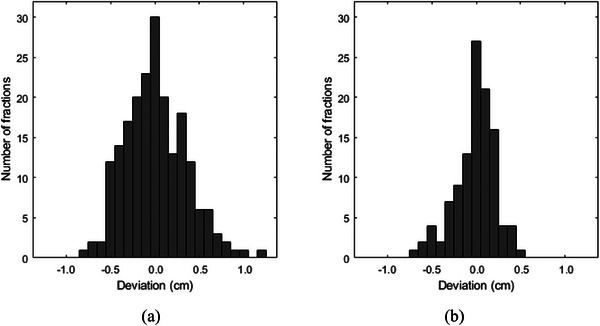
The daily deviation from the planned craniocaudal distance between the cranial and upper spinal isocenters (a) and the upper and lower spinal isocenters (b). The deviation is defined as the difference between the isocenter distance measured from daily setup images matched to the anatomy and the planned isocenter distance. Positive and negative deviations correspond to smaller and larger overlap between the fields of the adjacent isocenter groups, respectively, compared to the treatment plan.

A total of 204 fractions were analyzed. Since five of the patients had only two isocenters, the deviation in the distance between isocenters 2 and 3 was analyzed for 111 fractions. The craniocaudal isocenter distance deviations for all fractions are shown in Figure 2. In the following text, negative deviation corresponds to shorter isocenter distance and larger overlap between the fields of the adjacent isocenter groups compared to the treatment plan. Positive deviation corresponds to longer isocenter distance and smaller overlap between the fields of the adjacent isocenter groups. Group systematic error, standard deviation of the systematic error and standard deviation of the random error were −0.01, 0.22, and 0.26 cm for the deviation between isocenters 1 and 2, and −0.02, 0.18, and 0.14 cm for the deviation between isocenters 2 and 3. The maximum deviation between isocenters 1 and 3 in the patients with three isocenters was 1.4 cm.

**FIGURE 3:**
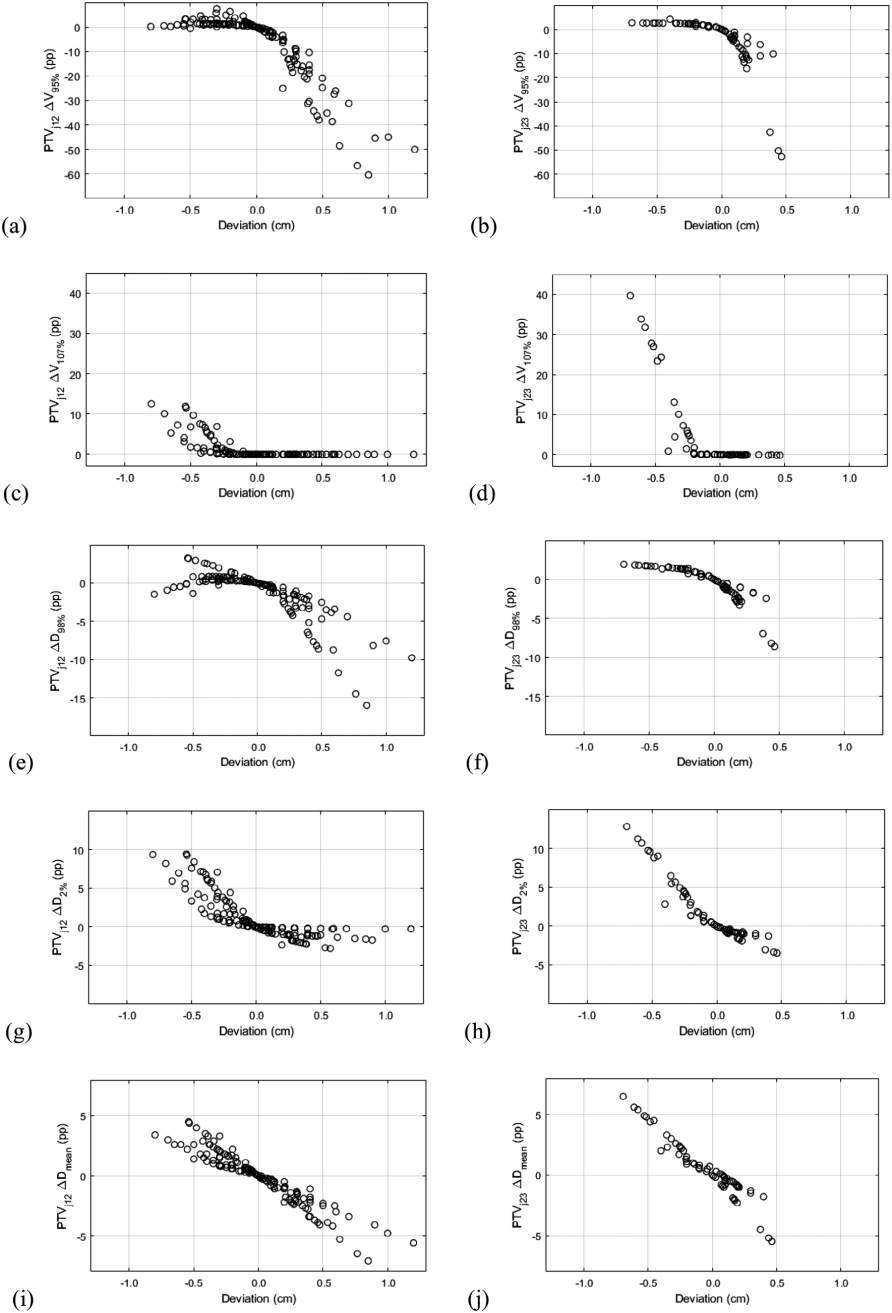
Effect of fraction‐level craniocaudal isocenter distance deviation on *V*_95%_ (a, b), *V*_107%_ (c, d), *D*_98%_ (e, f), *D*_2%_ (g, h) and *D*_mean_ (i, j) of the junction planning target volumes (PTV_j12_ in the left column and PTV_j23_ in the right column). Positive and negative deviations correspond to smaller and larger overlap between the fields of the adjacent isocenter groups, respectively, compared to the treatment plan. V_X%_ stands for the volume that is covered by the *X*% isodose, D_Y%_ for the dose that covers *Y*% of the structure and pp for percentage point.

The fraction‐level dosimetric effect of isocenter distance deviation on PTV_j12_ and PTV_j23_ is quantified in Figure 3. The effect of isocenter distance deviation on CTV_j12_ and CTV_j23_ was similar to the effect on PTV_j12_ and PTV_j23_ (Figure S1). In Figures S2 and S3, the original junction dose profiles and junction dose profiles are demonstrated after ±5 mm isocenter shifts of a representative patient's VMAT and 3D‐CRT plans.

For fraction‐level isocenter distance deviations ≤3 mm, *V*_95%_ of PTV_j12_ and PTV_j23_ decreased by more than 5 percentage points (pp) in 19% and 21% of fractions, respectively. For deviations ≤5 mm, *V*_95%_ of PTV_j12_ and PTV_j23_ decreased by more than 5 pp in 26% and 24% of fractions, respectively. For deviations ≤5 mm, *V*_107%_ of PTV_j12_ and PTV_j23_ increased by more than 10 pp in 0% and 5% of fractions, respectively. For deviations ≤3 mm, the changes in *D*_98%_ and *D*_2%_ were less than 5 pp except for a single fraction of one patient with 7.1 pp increase in *D*_2%_ of PTV_j12_. For deviations ≤ 5 mm, the conditions |Δ*D*_98%_| ≤5 pp and |Δ*D*_2%_| ≤ 5 pp would have been satisfied for more than 90% of fractions. For deviations ≤ 3 mm, the maximum |Δ*D*_mean_| was 3.3 pp. For deviations ≤ 5 mm, the maximum |Δ*D*_mean_| was 5.5 pp.

**FIGURE 4:**
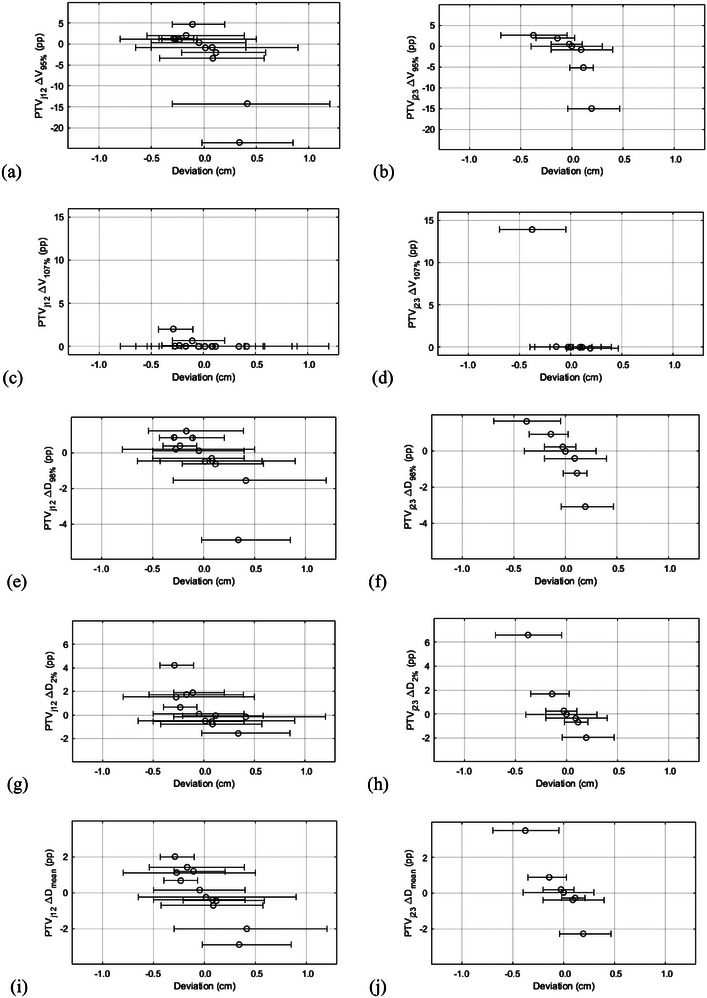
The cumulative effect of craniocaudal isocenter distance deviation on *V*_95%_ (a, b), *V*_107%_ (c, d), *D*_98%_ (e, f), *D*_2%_ (g, h), and *D*_mean_ (i, j) of the junction planning target volumes (PTV_j12_ in the left column and PTV_j23_ in the right column) for each patient. Positive and negative deviations correspond to smaller and larger overlap between the fields of the adjacent isocenter groups, respectively, compared to the treatment plan. V_X%_ stands for the volume that is covered by the *X*% isodose, D_Y%_ for the dose that covers *Y*% of the structure and pp for percentage point. The open circles indicate the mean setup error per patient, and the error bars indicate the minimum and maximum setup error.

The effect of isocenter distance deviations on the cumulative dose distribution is shown in Figure 4. The median (minimum, maximum) changes in *D*_mean_, *V*_95%_, *V*_107%_, *D*_98%_ and *D*_2%_ of PTV_j12_ of all patients were −0.1 (−2.9, 2.0) pp, −0.2 (−23.5, 4.7) pp, 0.0 (0.0, 2.0) pp, −0.1 (−4.9, 1.2) pp and 0.0 (−1.6, 4.2) pp, respectively. The median (minimum, maximum) changes in *D*_mean_, *V*_95%_, *V*_107%_, *D*_98%_, and *D*_2%_ of PTV_j23_ of all patients were 0.0 (−2.3, 3.5) pp, 0.0 (−15.0, 2.6) pp, 0.0 (−0.1, 13.9) pp, 0.0 (−3.1, 1.6) pp, and 0.0 (−2.0, 6.6) pp, respectively. The cumulative changes in *D*_mean_, *D*_98%_ and *D*_2%_ were less than 5 pp except for one patient with mean deviation of −4 mm between isocenters 2 and 3. For this patient, the cumulative deviation in *D*_2%_ of PTV_j23_ was 6.6 pp and the maximum point dose of PTV_j23_ was 117%, which corresponds to 35.8 Gy with the fractionation scheme that was used for the patient.

## 4 DISCUSSION

In this study, we reported that when adapting the isocenter distance to daily patient position in craniospinal irradiation using VMAT with low junction dose gradients, less than 5 pp cumulative change in the junction region *D*_98%_, *D*_2%_, and *D*_mean_ can be expected if the mean isocenter distance deviation over the treatment course is at most 3 mm. On fraction‐level, junction errors larger than 3 mm had more than 5 pp effect on the junction region *D*_98%_ and *D*_2%_ in several patients. However, random fraction‐level errors were seen to effectively cancel out over the whole treatment course.

The isocenter distance deviations that were observed in setup images of the patients in our study had similar magnitude to those reported in earlier studies.^6^, ^7^ The deviations between isocenters 1 and 2 had larger systematic and random errors compared to the deviations between isocenters 2 and 3. The position of the cervical spine is often difficult to reproduce accurately, which may explain the difference. The largest deviation between isocenters 1 and 3 was 14 mm in our study. If the treatment plan‐based fixed isocenter distance was used with such a large setup error, the craniocaudal PTV–CTV margin might not be enough to cover the setup error. This might lead to substantially reduced dose to the most cranial or caudal part of PTV.

International Commission on Radiation Units and Measurements has recommended a 5% limit for dose delivery errors.^25^, ^26^ Several studies assessing the dosimetric effect of setup errors have used 5% as an acceptable limit for dosimetric errors.^21^, ^27^, ^28^ In our study, limiting the allowed fraction‐level isocenter distance deviation at 3 mm would have resulted in less than 5 pp deviations in *D*_98%_ and *D*_2%_ except for a single fraction with 7.1 pp increase in *D*_2%_ of PTV_j12_. A 5 pp deviation in *D*_98%_ or *D*_2%_ does not exactly correspond to 5% of the prescribed dose; however, it is an adequate approximation. The maximum change in *D*_mean_ was 3.3 pp for deviations ≤3 mm, which we consider acceptable. However, the 5% limit for dose delivery errors should also include other sources for errors, such as linear accelerator output and dose calculation accuracy, which were not considered in this study.

The quality of the cumulative junction volume dose distribution was mainly determined by the mean isocenter distance deviation, that is, the systematic error, over the treatment course. Even though fraction‐level isocenter distance deviations higher than 3 mm had substantial dosimetric effect on the junction volume, the random deviations over the course of treatment cancelled each other out so that the cumulative dose distribution was acceptable in most cases except for one patient with mean deviation of −4 mm between isocenters 2 and 3. Even for this patient, the maximum point dose of the cumulative dose distribution was 35.8 Gy, which is far below 54 Gy, which is estimated to cause <1% risk of myelopathy with conventional fractionation of 2 Gy per day.^29^ Based on this, we conclude that isocenter distance deviation can be safely conducted in craniospinal irradiation using VMAT with the field arrangement presented in our study as long as the mean isocenter distance deviation over the treatment course is ≤3 mm. The problem is that the systematic isocenter distance deviation is difficult to predict. A potential approach could be to determine the mean isocenter distance deviation after the first half of the treatment course. If the deviation exceeds 3 mm, one should either limit the deviations from the planned isocenter distance during the second half of the treatment course or acquire a new planning image and make a new treatment plan.

The dosimetric effect of isocenter distance deviations on junction region CTV was similar to the effect on junction region PTV. The margin between CTV and PTV covers setup errors in lateral and anterior‐posterior directions. However, the PTV–CTV margin does not reduce the hot or cold spots caused by increased or decreased field overlap in the junction region CTV.

Even if the cumulative effect of isocenter distance deviations on the dose distribution was minimal, the random variation in the local dose per fraction could have a non‐negligible radiobiologic effect. Though not considered in this study, the radiobiologic effect of fraction‐level setup errors could be calculated by using the linear‐quadratic model.^30^, ^31^ Bortfeld and Paganetti^31^ reported that random dosimetric deviations with standard deviation of 10% lead to less than 1% error in the normalized total dose corrected for biologic effects of fraction‐level dosimetric variation. Thus, we assumed that the physical summation of the fraction‐level doses was sufficient to describe the cumulative dose distribution, and the radiobiologic effect of random variation in fraction‐level doses was negligible.

We simulated the isocenter shifts on the static anatomy of the planning CT image because only two‐dimensional setup images were available. We chose to shift the spinal isocenters and keep the cranial isocenter at its original location because of the shape of the treatment target. Shifting the cranial isocenter would have introduced dosimetric deviations at the base of the skull due to the large variation in target shape and body contour in craniocaudal direction at this anatomical location. Since the spinal canal is relatively cylindrical in shape, the dosimetric deviations related to the target size and shape variation were considered negligible with isocenter shifts in order of millimeters. For six patients, the junction region between isocenters 1 and 2 partially covered the skull. For three of these patients, slight dose minimum, corresponding to less than −1.5 pp change in *D*_98%_, was seen at the base of the skull with cranial shifts of isocenter 2. If the junction region is located at an anatomical location with large variation in the target shape and body contour, the junction region might be more sensitive to dosimetric hot or cold spots when the isocenter distance is adapted. For this reason, we recommend arranging the treatment fields so that the overlapping volume does not include the skull region.

The robustness of a craniospinal irradiation plan to isocenter distance deviations depends on the dose gradients at the field junction region. The most straightforward approach to optimizing the dose gradients is to overlap the fields over the whole junction region and let the optimization algorithm create the dose gradient without explicitly controlling the dose distribution.^21^, ^22^ This technique may result in high dose gradients at the edges of the junction region. Myers et al.^14^ and Strojnik et al.^15^ introduced a gradient optimization method, which produces a nearly linear staircase‐like dose gradient. The gradient optimization method is quite labor‐intensive because it requires manual contouring of optimization structures. Wang et al introduced a staggered overlap method and reported that it is equally robust towards isocenter distance deviations compared to the gradient optimization method.^16^ McVicar used a technique with *in‐silico* base dose plans, which produces a truly linear dose gradient and does not involve manual contouring of optimization structures.^18^ However, it involves using an in‐house‐made script and transferring data outside the treatment planning system. Our method of optimizing the field junctions is similar to the overlap and staggered overlap methods utilizing auto‐feathering described by Maddalo et al.^19^ They reported that the overlap and staggered overlap methods utilizing auto‐feathering had similar robustness to isocenter distance deviations as the gradient optimization method.

The dose gradients may be affected by the length of the overlap region. It has been reported that a shorter overlap region is correlated with larger hot and cold spots in the field junction region when isocenter distance deviations are introduced.^16^, ^19^ In our study, the average dose gradient, calculated based on the median overlap length, was 0.8%/mm and 0.9%/mm for the cranial and caudal junction, respectively. As shown in the dose gradient plot of a representative patient in Figure S2, the auto‐feathering algorithm effectively used the whole overlap length to create a relatively linear dose gradient. Still, there were local dose gradients higher than the average dose gradient. It is important to verify the shape of the dose gradients because some optimization algorithms may create steep local gradients despite long overlap length. We did not use constant overlap lengths for all patients because of their different sizes and anatomies. We aimed to use as long overlap region as possible considering the PTV size and the maximum field size. Wang et al^16^ reported that an overlap length of 9 cm was more optimal than 3 cm or 6 cm. Sarkar et al^21^ recommended overlapping the fields by at least 10 cm. The 7−20 cm overlap that we used should be long enough to produce a sufficiently low dose gradient. In addition to the overlap length, the dose gradients are also affected by the optimization algorithm, field arrangement, multileaf collimator width and optimization criteria.^16^

Using three isocenters instead of two might enable longer overlap regions, which would theoretically result in a lower dose gradient and more robust plan quality towards isocenter distance deviations. In addition, the setup deviations would be divided between three isocenters. Thus, the deviations between adjacent isocenters would be smaller compared to a plan with only two isocenters. However, using three isocenters increases the time and complexity of treatment delivery.

In our study, the data were limited to 12 patients because of the low number of craniospinal irradiation treatments conducted at our department. The number of patients is typical of studies on setup errors in craniospinal irradiation treatments.^7^, ^14^, ^15^, ^16^, ^17^, ^18^, ^19^ In addition, some of the patients only had one setup image per isocenter. However, a high number of fractions was included. The patients were treated during a 9‐year period. Thus, there was variation in the treatment procedures, such as fixation, fractionation and PTV–CTV margin. The image guidance procedure at our department at the time of data collection was not representative of image guidance protocols including cone‐beam CT and six‐degrees‐of‐freedom rotational correction. As the patient setup in craniospinal irradiation is based on bony structures, the measured isocenter distance would probably not be significantly different in cone‐beam CT images compared to planar setup images. Thus, using cone‐beam CT images would not significantly affect the results of our study. Six‐degrees‐of‐freedom treatment couch would enable accurate correction of rotational setup errors. Simulating the effect of rotational corrections on the junction dose distribution would be an interesting topic for a follow‐up study.

Another limitation of our study was that we only considered isocenter distance deviations in craniocaudal direction. The main reason for this was that orthogonal setup images were not available for the patients originally treated using 3D‐CRT. In addition, it has been reported that the dosimetric effects of lateral and anterior‐posterior junction setup errors are smaller than the effect of craniocaudal junction errors in craniospinal irradiation and total marrow irradiation using VMAT.^16^, ^32^, ^33^

A strength of our study was that the patient population included both pediatric and adult patients of different sizes and diagnoses. Thus, the results can be widely applied for craniospinal irradiation of different patient groups. The findings may also provide valuable insights into isocenter distance adaptation in other radiotherapy treatments requiring multiple isocenters, such as total marrow or total body irradiation.^34^, ^35^

## 5 CONCLUSIONS

Up to 12 mm deviations were observed in the craniocaudal distance between adjacent isocenters. The effects of fraction‐level isocenter distance deviations up to 3 mm on the junction dose distribution were acceptable. The cumulative dosimetric effect was acceptable except for one patient with mean isocenter distance deviation higher than 3 mm between the upper and lower spinal isocenters. Thus, we conclude that craniocaudal isocenter distance adaptation can be conducted provided that the mean isocenter distance deviation over the treatment course is at most 3 mm.

## AUTHOR CONTRIBUTIONS

Annele Heikkilä: contributed to study design, performed data analysis, wrote the manuscript, arranged funding. Antti Vanhanen: contributed to study conception and data interpretation, revised the manuscript. Maija Rossi: contributed to study conception and data interpretation, created the treatment plans, revised the manuscript. Tuomas Koivumäki: contributed to data interpretation, revised the manuscript. Michiel Postema: contributed to data interpretation, revised the manuscript. Eeva Boman: contributed to study conception and data interpretation, revised the manuscript. All authors approved the manuscript version to be published.

## CONFLICT OF INTEREST STATEMENT

The authors declare no conflicts of interest.

## Supporting information



Supporting Information
