# Supplementary material for: Dosimetric consequences of adapting the craniocaudal isocenter distance to daily patient position in craniospinal irradiation using volumetric modulated arc therapy
Source: J Appl Clin Med Phys. 2024 Oct 24;26(1):e14530. doi: 10.1002/acm2.14530 (PMC11713468; doi:10.1002/acm2.14530)
Supplement: Supplementary file 1 — Supporting Information [file ACM2-26-e14530-s001.docx]

# Supplementary material

**Dosimetric effect of fraction-level isocenter distance deviations on CTV_j12_ and CTV_j23_**

(a)
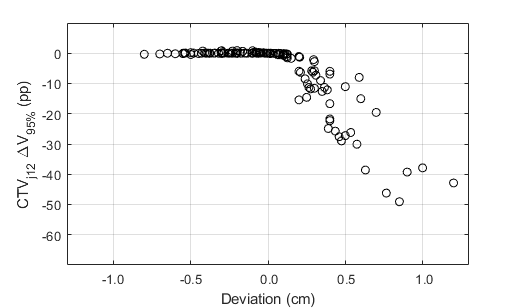
(b)
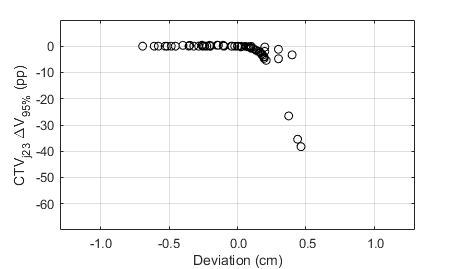


(c)
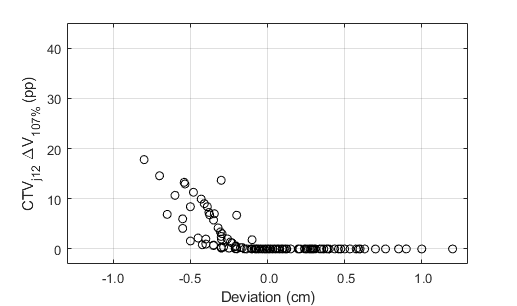
(d)
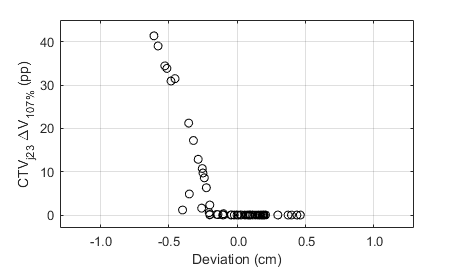


(e)
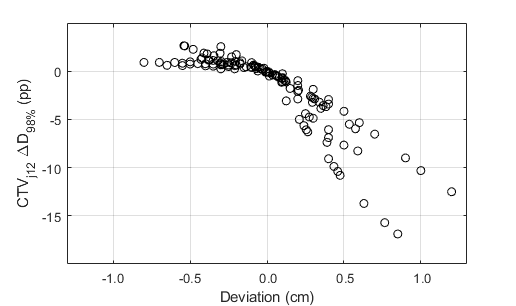
(f)
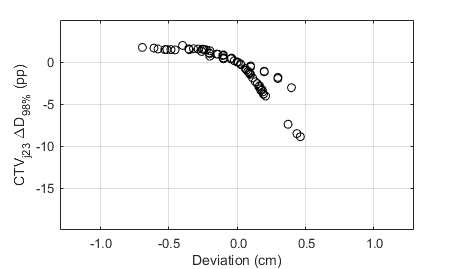


(g)
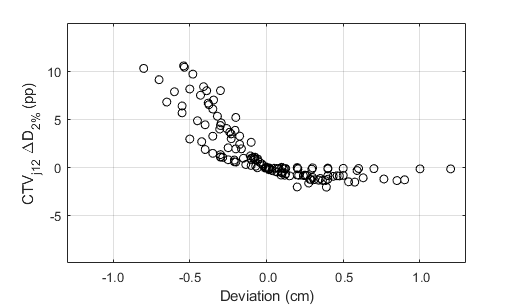
(h)
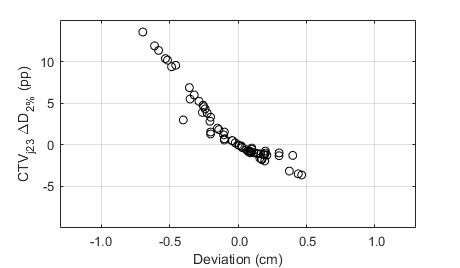


(i)
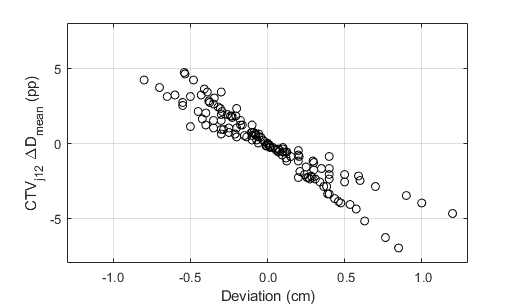
(j)
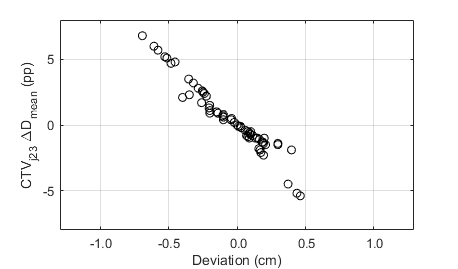


Figure 1 Effect of fraction-level craniocaudal isocenter distance deviation on V_95%_ (a-b), V_107%_ (c-d), D_98%_ (e-f), D_2%_ (g-h) and D_mean_ (i-j) of the junction clinical target volumes (CTV_j12_ in the left column and CTV_j23_ in the right column). V_X%_ stands for the volume that is covered by the X% isodose, D_Y%_ for the dose that covers Y% of CTV_j12_ and pp for percentage point.

**Junction profiles of 3D-CRT and VMAT plans**

A 3D-CRT plan was created for a representative patient to illustrate the effect of the anatomy-based isocenter shift on the dose distribution at the junction region using a 3D-CRT plan. The plan consisted of two isocenter groups with 6 MV lateral opposing brain fields and 18 MV posterior spine field. The collimator angles of the lateral fields were adjusted so that the caudal field edges matched to the divergence of the spinal field. Craniocaudal ±5 mm shifts of the isocenters with respect to each other were simulated by moving the cranial and caudal isocenters in opposite directions by 2 mm and 3 mm, respectively. Similar shifts were simulated for the VMAT plan of the same patient. Dose profiles along the junction region were obtained for the original and shifted 3D-CRT and VMAT plans. The isocenter group-specific dose profiles of the original plans are plotted in Figure 2. The dose profiles of the original and shifted 3D-CRT and VMAT plans are plotted in Figure 3. After +5 mm isocenter shift, the minimum dose to 1 cm^3^ of PTV_j12_ decreased by 48.5% in the 3D-CRT plan and by 9.4% in the VMAT plan. After -5 mm isocenter shift, the maximum dose to 1 cm^3^ of PTV_j12_ increased by 49.5% in the 3D-CRT plan and by 9.7% in the VMAT plan.

(a)
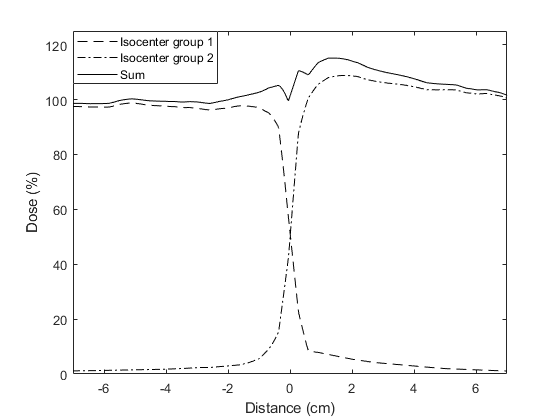
(b)
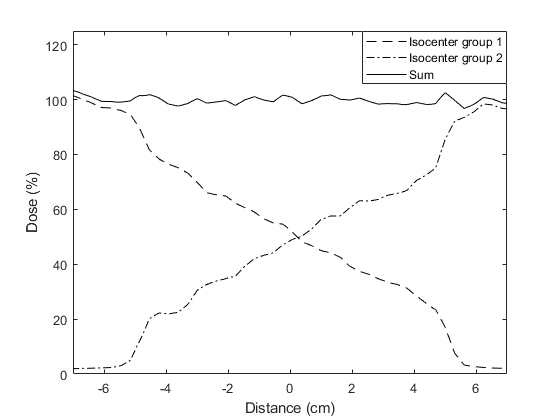


Figure 2 Isocenter group-specific dose profiles over the field overlap region of a representative patients’ 3D-CRT plan (a) and VMAT plan (b). The contribution of the fields of isocenter group 1 (cranial) and isocenter group 2 (caudal) are shown in a dashed line and a dash-dotted line, respectively. The total dose is shown in a solid line.


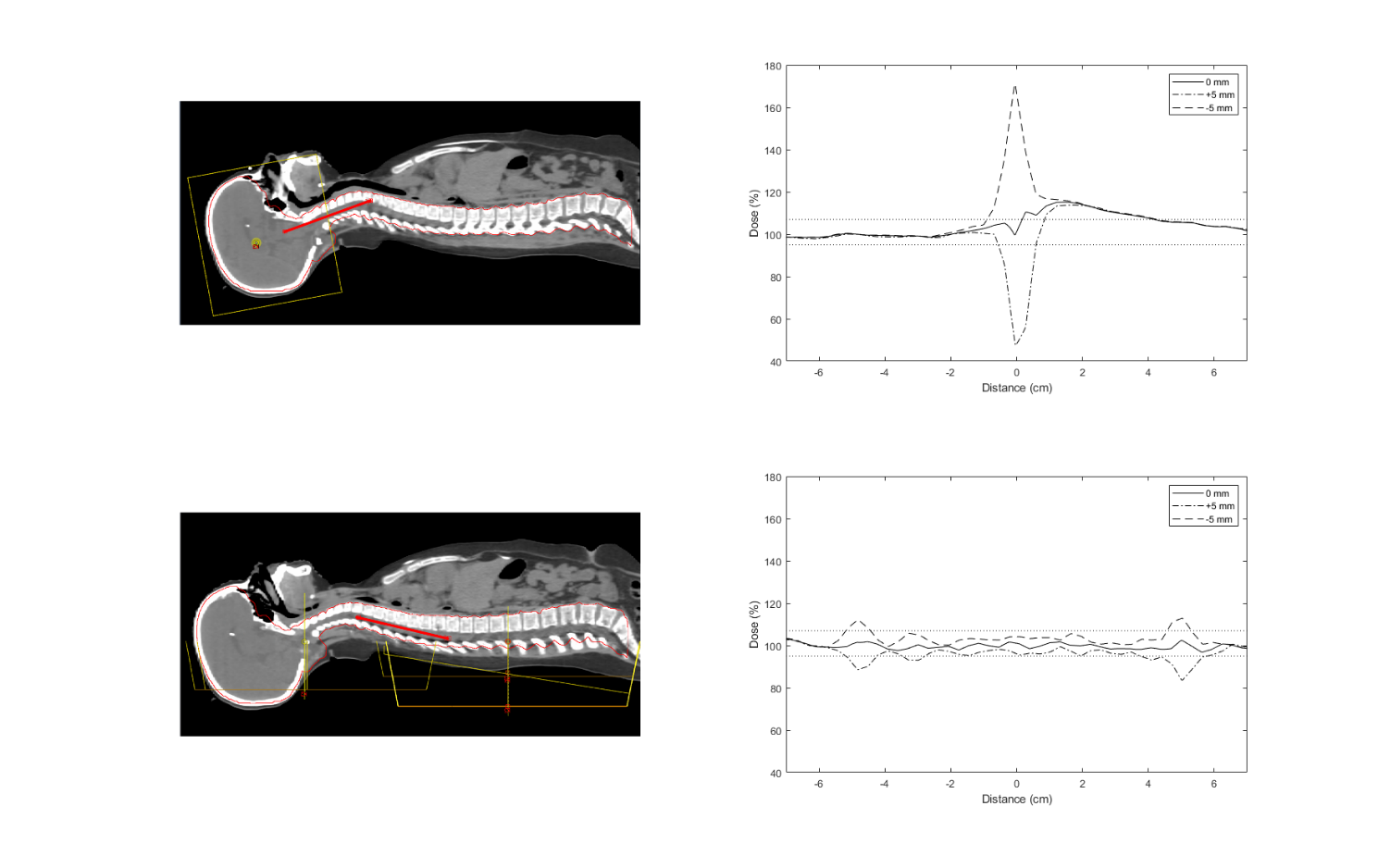


(a) (b)

(c) (d)

Figure 3 Dose profiles over the field junction for a representative patient’s original three-dimensional conformal radiotherapy (3D-CRT) and volumetric modulated arc therapy (VMAT) plans (solid line) and plans where the isocenters were shifted 5 mm away from (dash-dotted line) and towards each other (dashed line). The profiles for 3D-CRT plans were acquired from the anatomical location shown in (a) and the profiles for VMAT plans from the location shown in (c). The dose profiles of 3D-CRT plan are plotted in (b) and those of VMAT plan in (d). 95% and 107% dose levels are indicated with horizontal dotted lines.
